# A systematic method for comparing multimorbidity in national surveys

**DOI:** 10.1186/s13104-022-06164-3

**Published:** 2022-08-17

**Authors:** Rifqah Abeeda Roomaney, Brian van Wyk, Victoria Pillay-van Wyk

**Affiliations:** 1 Burden of Disease Research Unit, South African Medical Research Council, Francie van Zyl Drive, Cape Town, South Africa; 2 School of Public Health, University of the Western Cape, Robert Sobukwe Drive, Bellville, Cape Town, South Africa

**Keywords:** Multimorbidity, Disease patterns, Latent class analysis, Prevalence, South Africa

## Abstract

**Objective:**

Due to gaps in the literature, we developed a systematic method to assess multimorbidity using national surveys. The objectives of this study were thus to identify methods used to define and measure multimorbidity, to create a pre-defined list of disease conditions, to identify potential national surveys to include, to select disease conditions for each survey, and to analyse and compare the survey findings.

**Results:**

We used the count method to define multimorbidity. We created a pre-defined list of disease conditions by examining international literature and using local data on the burden of disease. We assessed national surveys, reporting on more than one disease condition in people 15 years and older, for inclusion. For each survey, the prevalence of multimorbidity was calculated, the disease patterns among the multimorbid population were assessed using a latent class analysis and logistic regression was used to identify sociodemographic and behavioural factors associated with multimorbidity. The prevalence of multimorbidity varied for each survey from 2.7 to 20.7%. We used a systematic and transparent method to interrogate multimorbidity in national surveys. While the prevalence in each survey differs, they collectively indicate that multimorbidity increases in older age groups and tends to be higher among women.

**Supplementary Information:**

The online version contains supplementary material available at 10.1186/s13104-022-06164-3.

## Introduction

Multimorbidity (the co-existence of a minimum of two long term disease conditions in one individual) is associated with a range of negative impacts, including a reduced quality of life [1], problems with medication adherence [2] and premature death [3]. There is a dearth of studies on multimorbidity in low and middle income countries (LMIC) [4]. While there is a growing research interest on multimorbidity in South Africa, the variability in survey methods led to disparate estimates on the prevalence of multimorbidity [5–7].

Several South African nationally representative surveys (e.g. South African Demographic and Health Survey [SAHDS], South Africa National HIV Prevalence, Incidence, Behaviour and Communication Survey [SABSSM], and the National Income Dynamics Study [NIDS]) provide important information about health conditions in the general population, particularly adults, and can be used to determine the prevalence and patterns of multimorbidity [6]. Information on the prevalence of disease clusters, trends and the characteristics associated with disease clusters present an opportunity to advocate for improved service delivery and target high-risk individuals. In the current paper, we illustrate a uniform method of analysing multiple national surveys to create a composite overview of multimorbidity disease prevalence and disease clustering and, compare findings of three nationally representative surveys in South Africa.

## Main text

### Methods

The objectives of this study were to: (a) identify methods used to define and measure multimorbidity, (b) create a pre-defined list of disease conditions to include in the study of multimorbidity, (c) identify potential national surveys to include, (d) select disease conditions for each survey, and (e) analyse and compare survey data (Additional file 1: Fig S1).

#### Multimorbidity measures and pre-defined disease condition list

The simplest and most common method to measure multimorbidity is to create an index—which is a count of the number of disease conditions in an individual using a predefined list of medical conditions [8, 9]. A multimorbidity variable can then be created by defining the number of people with two or more disease conditions as multimorbid. The type of disease conditions and the number of disease conditions included in studies of multimorbidity differ. A study recommended that disease conditions be included if they are commonly assessed in other multimorbidity studies or are relevant to the population under study [10]. Studies of multimorbidity have commonly included conditions such as hypertension (high blood pressure), chronic obstructive pulmonary disease (COPD), diabetes, malignancy, stroke, dementia, depression, joint disease, anxiety, congestive heart failure, coronary heart disease, asthma, cardiac arrhythmia, thyroid disease, anaemia, hearing problems, dyslipidemia, obesity, prostatic hypertrophy and osteoporosis [9–14]. We also reviewed the list of common disease conditions found in a mortality based study, the second South African National Burden of Disease Study (SANBD2) [15]. The SANBD2 list overlaps and differs with various conditions commonly included in other studies of multimorbidity (Additional file 1: Fig. S2). However, the SANBD2 also includes HIV, TB, diarrhoeal disease, lower respiratory infections and injuries as these are important to the South African burden of disease. We excluded acute conditions (diarrhoea and lower respiratory infections) and violence due to difficulty with measuring these conditions in a cross-sectional survey.

#### Survey inclusion

We searched online data repositories (e.g. DataFirst, Human Sciences Research Council, World Health Organization and Statistics South Africa) for potentially eligible surveys. Surveys were considered potentially eligible if they focused on South African adults and youth (people aged 15 years and older), were nationally representative, collected data post-1994 (after apartheid in South Africa) and contained relevant information (i.e. allow for the calculation of multimorbidity prevalence). We also considered the methodological quality of the surveys (e.g. methodological issues specific to each survey such as survey skip patterns, differences in target population and sampling strategies, response rates, and the way in which sampling weights have been calculated and calibrated to population totals).

Potentially eligible datasets were downloaded from data repositories and data user agreements were accepted. Data user agreements were saved to an electronic file. Due to the number of surveys deemed eligible, we focused on the most recent set of surveys.

#### Survey details and disease conditions

Data were extracted from each survey regarding the survey’s study design, sampling and the variables of interest. Disease conditions were assessed against the pre-defined lists of disease conditions. We noted how the disease conditions of interest were measured (i.e. self-reported or physically measured). For example, if blood pressure was physically measured, the instrument used, and the number of repeated measurements were recorded.

Where disease conditions were self-reported, the survey questions were documented in Microsoft Excel. We included self-reported disease conditions that were “current” at the time of the survey. Disease conditions were excluded if the condition could not be assumed to be current due to the way the question was asked. For example, if the participant was asked if they have ‘ever had cancer’, it could not be assumed that they had cancer at the time of the survey. In certain cases, it was appropriate to include diseases where the participant was asked whether they had ‘ever’ been diagnosed with the disease, such as in the case with a chronic disease with minimal chances of cure (e.g. HIV).

#### Other variables of interest

Sociodemographic and behavioural data that could be associated with multimorbidity—such as age, sex, educational attainment, employment status, socioeconomic status, locality, alcohol and tobacco consumption, and information on body mass index—were extracted. These variables were identified based on an overview of five systematic reviews that identified biomedical, socioeconomic, social and environmental, and behaviours associated with multimorbidity [13].

#### Data analysis

Data analysis consisted of three main components which was to estimate the prevalence of multimorbidity by age and sex, identify characteristics associated with multimorbidity using a logistic regression and latent class analysis to identify disease clusters or classes within the multimorbid population. The logistic regression and latent class analysis are described in detail in Roomaney et al. [6, 7]. All survey datasets were weighted to the South African population using Statistics South Africa data for the appropriate year. All results shown are weighted.

### Results

Three surveys were selected due to these being the most recent health-related, nationally representative surveys in South Africa. Additional file 1: Table S1 describes the various aims and methods employed by each survey (e.g. survey design, sampling methods and data access). SADHS 2016 and SABSSM 2017 used similar survey methods.

Additional file 1: Table S2 shows the disease conditions included in each survey. Between four and nine disease conditions were investigated per survey (i.e. SADHS 2016 = 9, SABSSM 2017 = 6 and NIDS 2017 = 4). All three surveys included diabetes, heart disease and hypertension; while HIV and TB were assessed in two surveys (SADHS 2016 and SABSSM 2017), and stroke was assessed in SADHS 2016 and NIDS 2017. SADHS 2016 measured HbA1c using dry blood spots to determine diabetes status. Similarly, HIV status was also determined via testing of a dry blood spot in SADHS 2016 and SABSSM 2017. Hypertension was measured using blood pressure monitors in SADHS 2016 and NIDS 2017. Additional file 1: Table S3 shows the prevalence of each disease in the surveys.

**Fig. 1:**
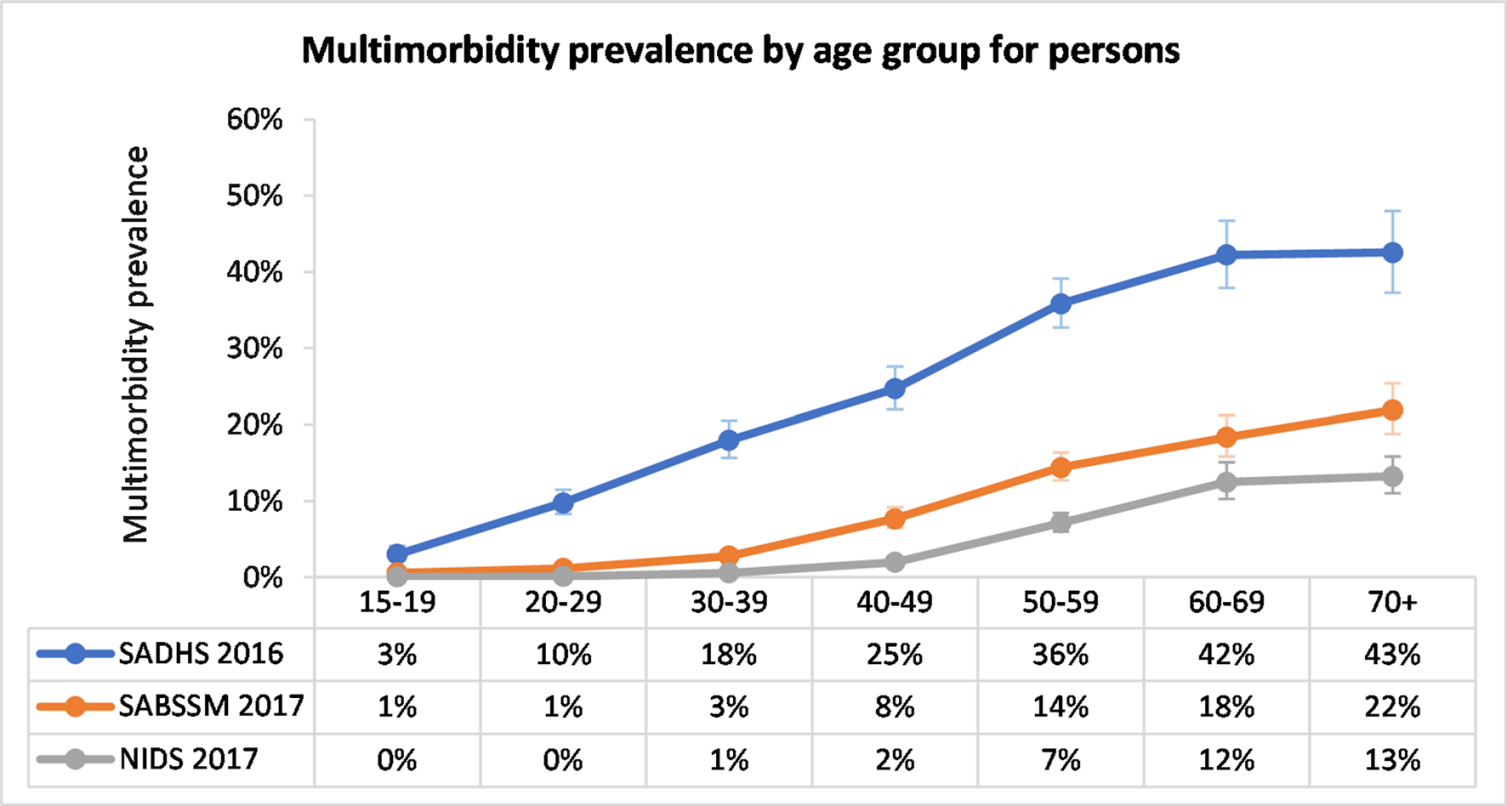
Prevalence of multimorbidity by age group and survey (weighted)

Table 1 illustrates the variability in the prevalence(s) of multimorbidity across the surveys. The calculated multimorbidity prevalence was highest in SADHS 2016 (20.7%); while 5.9% and 2.7% calculated for SABSSM 2017 and NIDS 2017, respectively. In each survey, the prevalence of multimorbidity was almost double in women compared to men. While the prevalence varied between the surveys, the pattern of multimorbidity by age group was similar—starting with a low prevalence and increasing as age increases (Fig. 1, Additional file 1: Table S4).

**Table 1:** Overall multimorbidity prevalence (weighted)

Number of disease conditions	SADHS 2016 (%, 95% CI)			SABSSM 2017 (%, 95% CI)			NIDS 2017 (%, 95% CI)		
	Total	Male	Female	Total	Male	Female	Total	Male	Female
No disease	48.6 (47.0–50.1)	55.8 (53.5–58.1)	41.8 (40.0–43.4)	71.9 (70.8–73.1)	78.6 (77.3–79.9)	65.9 (64.3–67.4)	74.5 (73.5–75.4)	76.0 (74.5–77.5)	71.7 (73.0–74.2)
1 disease	30.8 (29.5–32.0)	29.4 (27.5–31.2)	32.1 (30.6–33.7)	22.2 (21.2–23.2)	17.3 (16.1–18.5)	26.7 (25.4–28.0)	22.8 (21.8–23.8)	22.1 (20.7–23.6)	22.3 (23.4–24.6)
2 diseases	14.1 (13.2–15.1)	10.5 (9.4 – 11.8)	17.4 (16.2–18.7)	4.9 (4.5–5.4)	3.5 (3.0–4.0)	6.3 (5.7–6.9)	2.3 (2.1–2.6)	1.5 (1.2–1.9)	2.7 (3.1–3.5)
3 + diseases	6.6 (5.9– 7.3)	4.3 (3.5–5.2)	8.7 (7.8–9.8)	0.9 (0.7–1.2)	0.6 (0.4–0.9)	1.2 (1.0–1.5)	0.4 (0.3–0.5)	0.3 (0.2–0.5)	0.4 (0.5–0.7)
Multimorbidity (≥ 2 diseases)	20.7 (19.5–21.9)	14.8 (13.4–16.3)	26.2 (24.7–27.7)	5.9 (5.4–6.4)	4.1 (3.6–4.7)	7.5 (6.8–8.2)	2.7 (2.4–3.1)	1.8 (1.5–2.3)	3.6 (3.2–4.0)

SADHS 2016: South African Demographic and Health Survey 2016. SABSSM 2017: South African National HIV Prevalence, Incidence, Behaviour and Communication Survey 2017. NIDS 2017: National Income Dynamics Study 2017

The surveys described different disease conditions, and therefore direct comparison of disease patterns is limited. However, as indicated in Table 2, the combination of Diabetes and Hypertension was prevalent in all three surveys, while heart disease and Hypertension was prevalent in two surveys. Hypertension was prominent in 8 out of 11 disease classes.

**Table 2:** Disease classes per survey

Surveys	SADHS 2016	SABSSM 2017	NIDS 2017
HIV, hypertension and anaemia	X		
Anaemia and hypertension	X		
Cardiovascular	X		
Diabetes and hypertension	X	X	X
HIV and hypertension		X	
Heart disease and hypertension		X	X
HIV, diabetes and heart disease		X	
TB and HIV		X	
Hypertension, TB and cancer		X	
All diseases except HIV		X	
Stroke and hypertension			X

The factors associated with multimorbidity varied between the surveys (Additional file 1: Tables S5 and S6). Older age was the most consistent factor associated with increased multimorbidity in all three surveys. Other sociodemographic factors that indicated an increased risk for multimorbidity was being female and living in an urban environment (in SABSSM 2017) and belonging to the wealthiest quintile (in NIDS 2017). Lifestyle factors associated with an increased risk of multimorbidity were being a smoker and having a high body mass index (both in NIDS 2017).

Level of education and employment status were associated with decreased odds of multimorbidity e.g., secondary and being employed (in SADHS 2016 and SABSSM 2017) and tertiary education (in NIDS 2017). Alcohol use was associated with decreased odds of multimorbidity in one survey (SADHS 2016)—which is may be linked to the ‘sick quitter’ hypothesis, i.e. sick people abstain from drinking alcohol due to taking prescribed medication which could lead to negative interactions [16].

### Discussion

In this paper we developed and used a systematic strategy to analyse multimorbidity prevalence and disease patterns in three national surveys. Several studies have highlighted the problematic variation in study design when assessing multimorbidity [17–20]. We followed recommendations of Nguyen et al. [17] to determine the prevalence of multimorbidity using a standardised protocol and to report multimorbidity by age and sex. This systematic method offers a way in which other LMIC can determine multimorbidity from available national survey data sets in the absence of robust routine health information. Our developed method allows for transparency in recording the survey differences and thus produces improved comparison between studies, particularly by reporting prevalence by age and sex using standardised intervals.

Two key findings were that multimorbidity was consistently higher among women compared to men; and that multimorbidity increased in older age groups. Although female sex has inconsistently been linked to higher levels of multimorbidity in South Africa [5], the findings on age and sex [17] are consistent with much of the international literature [21]. Rising multimorbidity in aging populations has implications for healthcare costs and service utilisation in a country such as South Africa with an ageing population [22].

Even though the surveys assessed different disease conditions, hypertension and diabetes was a disease combination common to all three surveys. Hypertension was involved with almost all the multimorbid disease patterns, whether it was combined with communicable or NCDs. At a minimum, this indicates the urgent need to regularly screen for hypertension in the adult population; particularly in those already diagnosed with a chronic disease. The management of co-occurring diseases, especially in the elderly, needs to be managed in an integrated manner to ensure optimal care.

## Conclusion and recommendations

We provided a systematic and transparent method that can be used to interrogate multimorbidity in national surveys. While the prevalence in each survey differs, they collectively indicate that multimorbidity increases in older age groups and tends to be higher in women. This is an important consideration to ensure equitable and efficient health service delivery in South Africa.

We recommend that future surveys ask self-reported questions in a consistent manner that can be used to analyse multimorbidity. We would also recommend that a consistent and minimum set of diseases are asked about in self-reported health questionnaires. This could be based on international surveys but also diseases that are important locally.

## Limitations

There were several limitations, most of which led to an under-estimation in disease prevalence. Firstly, each survey had a different amount of disease conditions available to analyse. In addition, the same disease conditions were not available in each survey hence this makes comparison of the prevalence of multimorbidity difficult.

We included self-reported disease conditions which may underestimate the prevalence as people may have been unaware that they have the disease. However, a recent systematic review indicated no significant difference in the prevalence of multimorbidity when self-report versus clinic/administrative data were used [21]. Where self-reported disease conditions were included, the way in which the question was asked at times differed. We excluded disease conditions that we could not confirm were current diseases. This would have also underestimated the prevalence of multimorbidity. We also excluded acute disease conditions.

## Supplementary Information

**Additional file 1: Fig. S1.** Study process flow. **Fig. S2.** Overlapping disease conditions. **Fig. S3.** Prevalence of multimorbidity by age group and survey (weighted). **Table S1.** Summary overview of included surveys. **Table S2.** A. Disease conditions by survey and method of measurement. B. Self-reported questions in each survey. **Table S3.** Prevalence of each disease condition by survey (weighted). **Table S4.** Prevalence of multimorbidity by age group and sex (weighted). **Table S5.** SABSSM 2017 Regression. **Table S6.** Factors associated with multimorbidity in adjusted models

## Data Availability

The SADHS 2016 datasets supporting the conclusions of this article are available upon request in the DHS Programme repository, [https://dhsprogram.com/data/dataset/South-Africa_Standard-DHS_2016.cfm?flag=0]. The SABSSM 2017 dataset supporting the conclusions of this article is available upon request in the Human Sciences Research Council Research Data Service repository, [https://doi.org/10.14749/1585345902 and https://repository.hsrc.ac.za/handle/20.500.11910/15468]. The NIDS 2017 Wave 5 dataset supporting the conclusions of this article are available upon request in the DataFirst repository, [https://doi.org/10.25828/fw3h-v708 and https://www.datafirst.uct.ac.za/dataportal/index.php/catalog/712].

## Abbreviations

COPD: Chronic obstructive pulmonary disease
HIV: Human immunodeficiency virus
NIDS: National Income Dynamics Study
SABSSM: South African National HIV Prevalence, Incidence, Behaviour and Communication Survey
SADHS: South African Demographic and Health Survey
SANBD2: Second South African National Burden of Disease Study
TB: Tuberculosis
