# Supplementary material for: A systematic method for comparing multimorbidity in national surveys
Source: BMC Res Notes. 2022 Aug 17;15:280. doi: 10.1186/s13104-022-06164-3 (PMC9387001; doi:10.1186/s13104-022-06164-3)
Supplement: Supplementary file 1 — Additional file 1: Fig. S1. Study process flow. Fig. S2. Overlapping disease conditions. Fig. S3. Prevalence of multimorbidity by age group and survey (weighted). Table S1. Summary overview of included surveys. Table S2. A. Disease conditions by survey and method of measurement. B. Self-reported questions in each survey. Table S3. Prevalence of each disease condition by survey (weighted). Table S4. Prevalence of multimorbidity by age group and sex (weighted). Table S5. SABSSM 2017 Regression. Table S6. Factors associated with multimorbidity in adjusted models [file 13104_2022_6164_MOESM1_ESM.docx]

**Additional file 1**


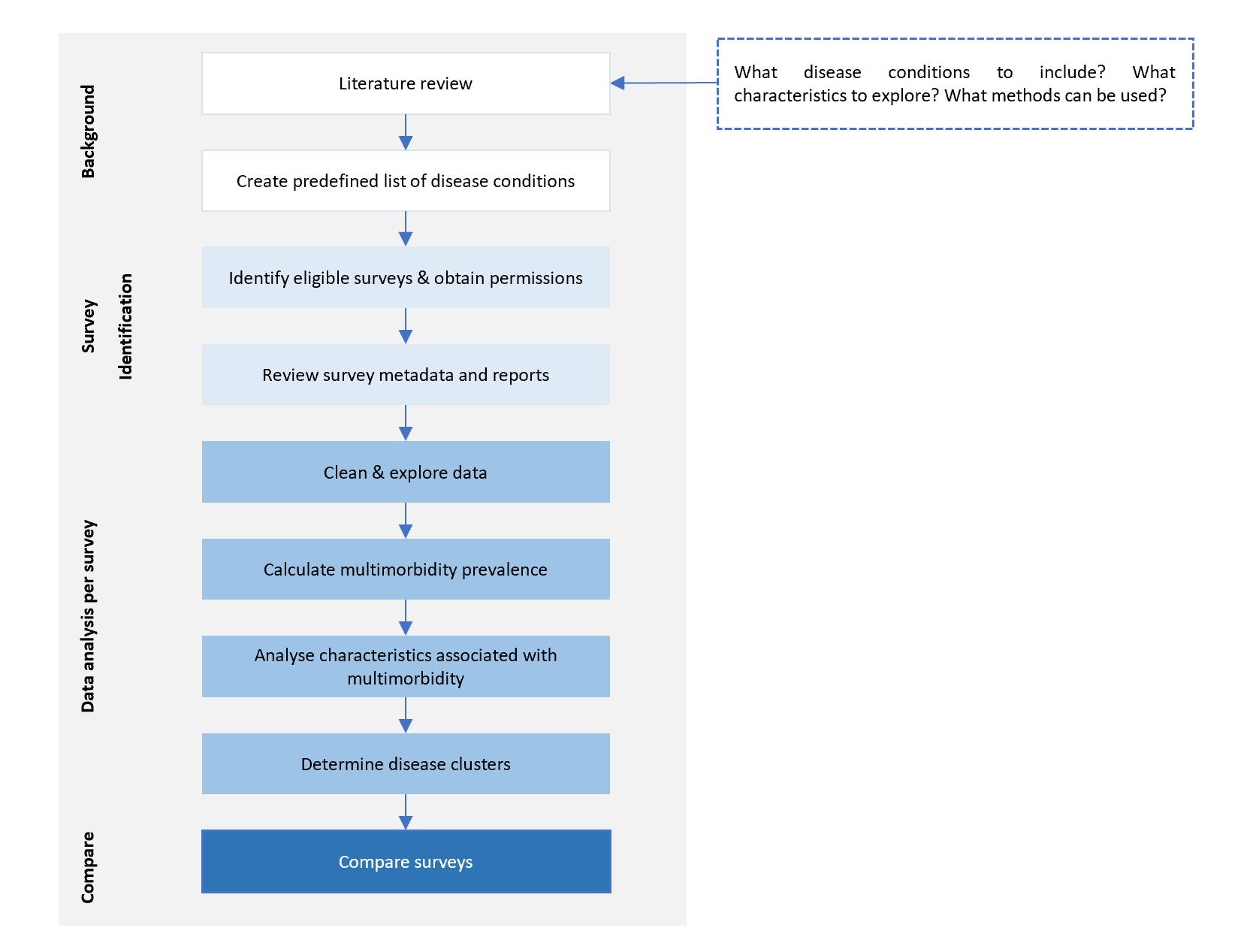


**Fig. S1. Study process flow.**


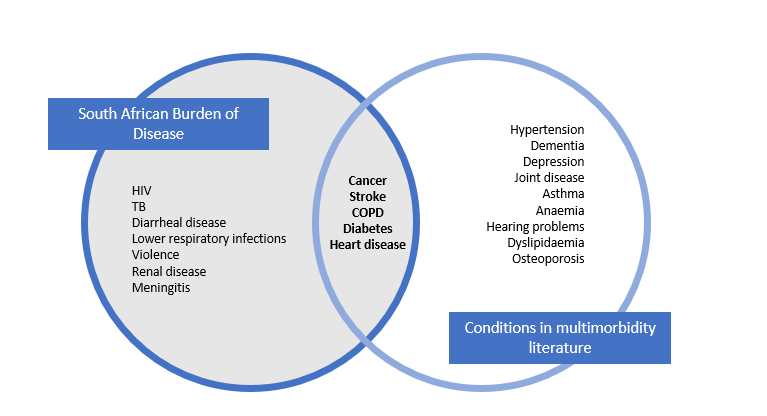


**Fig. S2. Overlapping disease conditions**

**Fig.S3. Prevalence of multimorbidity by age group and survey (weighted)**

**Table S1. Summary overview of included surveys**

| **Surveys** | **SADHS 2016** | **SABSSM 2017** | **NIDS 2017** |
| --- | --- | --- | --- |
| Previous surveys | 2003, 2008. | 2002, 2005, 2008, 2012. | 2008, 2010, 2012, 2014. |
| Survey aim | Provide up-to-date estimates of basic demographic and health indicators. | Determine the HIV status and  exposure to antiretroviral medicine (ARV) among South Africans. | Examine the livelihoods of individuals and households over time. |
| Survey design | National cross-sectional household survey. | National cross-sectional household survey. | National household panel (longitudinal) survey. |
| Sampling | Multistage cluster random sample (stratified two-stage sample design). | Multistage stratified random cluster sampling. | Study started in 2008 with a nationally representative sample and was repeated with the same sample every two years. People who became part of the household were also interviewed. A Top-up sample was added in 2017 due to sample attrition. |
| Master sampling frame | Statistics South Africa Master Sample Frame (created using Census 2011 enumeration areas). | Statistics South Africa 2015 national population sampling frame consisting of 84 907 small area layers (SALs). |  |
| Primary sampling unit | Enumeration area. | 1000 SALs from 2015 database of SALs. |  |
| Household selection | All dwelling units (households) in an enumeration area were listed and 20 dwelling units were systematically selected. | 15 visiting points were systematically sampled from 1000 SALs. |  |
| Date of data collection | 27 Jun - 4 Nov 2016. | Dec 2016 – Feb 2018. | Feb – Dec 2017. |
| Access to survey | Anonymised dataset available from DHS Project. | Anonymised dataset available from Human Sciences Research Council. | Anonymised dataset available from DataFirst. |
| Funding sources | NDoH, SAMRC, Global Fund, EU, UNICEF, UNFPA, ICF. | PEPFAR, CDC, Department of Science and Technology, South African National AIDS Council,  Global Fund, Right to Care, UNICEF, USAID, Soul City and loveLife. | Department of Planning, Monitoring and Evaluation. |

SADHS 2016: South African Demographic and Health Survey 2016. SABSSM 2017: South African National HIV Prevalence, Incidence, Behaviour and Communication Survey 2017. NIDS 2017: National Income Dynamics Study 2017.

**Table S2.A. Disease conditions by survey and method of measurement**

| **Disease condition** | **SADHS 2016** | **SABSSM 2017** | **NIDS2017** | **No. included** |
| --- | --- | --- | --- | --- |
| **Diabetes** | Measured | Self-reported | Self-reported | 3 |
| **Heart disease** | Self-reported | Self-reported | Self-reported | 3 |
| **Hypertension** | Measured | Self-reported | Measured | 3 |
| **HIV** | Measured | Measured | - | 2 |
| **Stroke** | Self-reported | - | Self-reported | 2 |
| **TB** | Self-reported (in the last 12 months) | Self-reported | - | 2 |
| **Anaemia** | Measured | - |  | 1 |
| **Cancer** | - | Self-reported | - | 1 |
| **COPD*** | Self-reported | - | - | 1 |
| **High blood cholesterol** | Self-reported | - | - | 1 |

**Table S2.B. Self-reported questions in each survey**

| **Disease condition** | **SADHS 2016** | **SABSSM 2017** | **NIDS 2017** |
| --- | --- | --- | --- |
| **Diabetes** | Has a doctor, nurse or health worker told you that you have or have had any of the following conditions: diabetes or blood sugar? | Do you currently have any of the following illnesses? (Diagnosed with illness) Diabetes | Have you ever been told by a doctor, nurse or health care professional that you have Diabetes or high blood sugar? |
| **Heart disease** | Has a doctor, nurse or health worker told you that you have or have had any of the following conditions: Heart attack or angina/chest pains? | Do you currently have any of the following illnesses? (Diagnosed with illness) Heart disease | Have you ever been told by a doctor, nurse or health care professional that you have heart problems? |
| **Hypertension** | Measured | Do you currently have any of the following illnesses? (Diagnosed with illness) Hypertension / high blood pressure | Measured |
| **HIV** | Measured | Measured | - |
| **Stroke** | Has a doctor, nurse or health worker told you that you have or have had any of the following conditions: stroke? | - | Have you ever been told by a doctor, nurse or health care professional that you have Stroke? |
| **TB** | Has a doctor, nurse or health worker ever told you that you have TB? When was the last time you had TB? | Do you currently have any of the following illnesses? (Diagnosed with illness) Tuberculosis/ TB | - |
| **Anaemia** | Measured | - | - |
| **Cancer** | - | Do you currently have any of the following illnesses? (Diagnosed with illness) Cancer | - |
| **COPD*** | Has a doctor, nurse or health worker told you that you have or have had any of the following conditions: chronic bronchitis, emphysema, or COPD? | - | - |
| **High blood cholesterol** | Has a doctor, nurse or health worker told you that you have or have had any of the following conditions: high blood cholesterol or fats in the blood? | - | - |

*Emphysema/ Bronchitis/COPD. Blue shade indicates that the disease condition was physically measured. SADHS 2016: South African Demographic and Health Survey 2016. SABSSM 2017: South African National HIV Prevalence, Incidence, Behaviour and Communication Survey 2017. NIDS 2017: National Income Dynamics Study 2017.

**Table S3. Prevalence of each disease condition by survey (weighted)**

| **Disease condition** | **SA DHS 2016** | | | **SABSSM 2017** | | | **NIDS 2017** | | |
| --- | --- | --- | --- | --- | --- | --- | --- | --- | --- |
|  | **Total** | **Males** | **Females** | **Total** | **Males** | **Females** | **Total** | **Males** | **Females** |
| Anaemia | 24.7  (23.2-26.3) | 16.8  (15.0-18.8) | 31.3  (29.2-33.5) | - | - | - | - | - | - |
| COPD* | 1.4  (1.1–1.8) | 1.1  (0.8–1.6) | 1.7  (1.3–2.2) | - | - | - | - | - | - |
| Cancer | - | - | - | 0.6  (0.5-0.8) | 0.4  (0.3-0.7) | 0.8  (0.6-1.1) | - | - | - |
| Cholesterol | 3.5  (2.9–4.2) | 3  (2.3–3.8) | 4.1  (3.4–4.9) | - | - | - | - | - | - |
| Diabetes | 9.1  (8.4–9.9) | 6.9  (5.9–7.9) | 11.1  (10.2–12.3) | 4.4  (4.0 - 4.9) | 3.7  (3.2 - 4.3) | 5.0  (4.5 - 5.6) | 2.9  (2.6-3.3) | 2.1  (1.7-2.5) | 3.3  (3.8-4.3) |
| Heart disease | 3.1  (2.7 – 3.5) | 2.3  (1.8 – 2.9) | 3.8  (3.3 – 4.5) | 2.2  (1.8 - 2.5) | 1.6  (1.3 - 2.1) | 2.6  (2.1 - 3.2) | 1.6  (1.3-1.8) | 1.2  (0.9-1.5) | 1.9  (1.6-2.3) |
| HIV | 19.6  (18.2–21.1) | 13.7  (11.8–15.8) | 24.5  (22.7–26.4) | 19.1  (17.9-20.4) | 14.3  (12.9-15.9) | 23.1  (21.7-24.7) | - | - | - |
| Hypertension | 45  (43.1– 46.9) | 44.1  (41.5– 46.7) | 45.8  (43.7–48.0) | 14.3  (13.5-15.2) | 10.4  (9.4 - 11.5) | 17.8  (16.7- 19.0) | 27.8  (26.7-29.0) | 27.3  (25.6-29.0) | 28.4  (27.1-29.7) |
| Stroke | 1.4  (1.1 – 1.7) | 1  (0.7 – 1.5) | 1.7  (1.4 – 2.1) | - | - | - | 0.8  (0.6-1.0) | 0.7  (0.5-0.9) | 0.9  (0.7-1.1) |
| TB | 1.2  (0.9 – 1.5) | 0.9  (0.6 – 1.3) | 1.5  (1.0 – 2.0) | 1.2  (1.0 - 1.4) | 1.3  (1.0 - 1.8) | 1.0  (0.8 - 1.4) | - | - | - |

* Emphysema/ Bronchitis/COPD. Blue highlight indicates the disease was measured (not self-reported)

**Table S4. Prevalence of multimorbidity by age group and sex (weighted)**

| **Age group** | **SA DHS 2016** | | | **SABSSM 2017** | | | **NIDS 2017** | | |
| --- | --- | --- | --- | --- | --- | --- | --- | --- | --- |
|  | **Total** | **Males** | **Females** | **Total** | **Males** | **Females** | **Total** | **Males** | **Females** |
| 15-19 years | 3.0  (2.2-4.1) | 1.9  (1.1-3.0) | 4.4  (3.0-6.5) | 0.6  (0.3-1.1) | 0.6  (0.2-1.6) | 0.5  (0.2-1.3) | 0.1  (0.0-0.2) | 0.0  (0.0-0.0) | 0.1  (0.0-0.4) |
| 20-29 years | 9.8  (8.3-11.5) | 4.8  (3.5-6.6) | 14.9  (12.3-17.8) | 1.2  (0.8-1.7) | 0.7  (0.3-1.6) | 1.6  (1.0-2.4) | 0.1  (0.0-0.3) | 0.0  (0.0-0.2) | 0.2  (0.1-0.5) |
| 30-39 years | 18.0  (15.6-20.5) | 11.2  (8.2-15.1) | 24.6  (21.5-28.0) | 2.8  (2.2-3.6) | 1.5  (1.0-2.4) | 4.0  (3.0-5.4) | 0.6  (0.3-1.0) | 0.5  (0.1-1.4) | 0.7  (0.4-1.2) |
| 40-49 years | 24.7  (22.0-27.6) | 18.5  (14.8-22.8) | 30.2  (26.7-34.0) | 7.7  (6.4-9.2) | 5.3  (3.8-7.5) | 9.9  (8.0-12.1) | 2.0  (1.5-2.6) | 1.3  (0.7-2.3) | 2.7  (1.9-3.6) |
| 50-59 years | 35.8  (32.7-39.1) | 31.9  (26.8-37.5) | 38.6  (34.7-42.8) | 14.4  (12.7-16.3) | 11.7  (9.2-14.8) | 16.6  (14.3-19.2) | 7.1  (6.0-8.4) | 4.9  (3.4-6.9) | 9.0  (7.4-10.9) |
| 60-69 years | 42.2  (37.9-46.7) | 36.6  (30.2-43.6) | 47.2  (42.3-52.1) | 18.4  (15.8-21.2) | 14.0  (10.7-17.9) | 21.7 (  18.1-25.9) | 12.5  (10.3-15.1) | 10.0  (7.1-13.7) | 14.4  (11.6-17.7) |
| 70+ years | 42.5  (37.3-48.0) | 39.7  (32.0-47.9) | 44.3  (38.3-50.4) | 21.9  (18.8-25.4) | 18.9  (14.2-24.8) | 23.7  (19.6-28.4) | 13.2  (11.0-15.8) | 11.5  (8.4-15.5) | 14.2  (11.5-17.4) |

**Table S5. SABSSM 2017 Regression**

| Variable | Unadjusted  Odds Ratios (95% CI) | Adjusted  Odds Ratios (95% CI) |
| --- | --- | --- |
| Age category (*Reference category: 15 -24 years*) | | |
| 25-34 | 5.2 (2.9-9.5)* | 5.8 (2.6-13.1) * |
| 35-44 | 10.2 (5.6-18.8)* | 11.3 (5.0-25.4) * |
| 45-54 | 28.5 (16.2-50.1)* | 30.8 (13.9-68.3) * |
| 55-64 | 43.3 (24.3-77.2)* | 41.5 (18.7-91.8) * |
| 65+ | 66.6 (37.6-117.9)* | 53.1 (23.9-117.7) * |
| Female (*Reference: Male)* | 1.9 (1.6-2.2)* | 1.7 (1.4-2.0) * |
| Urban location *(Reference: Rural)* | 1.6 (1.3-1.9) * | 1.9 (1.5-2.3) * |
| Education *(Reference: Primary)* |  |  |
| Secondary | 0.4 (0.4-0.5) * | 0.9 (0.7-1.1) |
| Tertiary | 0.4 (0.3-0.6) * | 0.7 (0.4-1.0) * |
| Employed *(Reference: Unemployed)* | 0.6 (0.5-0.7) * | 0.5 (0.4-0.7) * |
| Current alcohol *(Reference: No drinking)* | 0.7 (0.6-0.9) * | 0.9 (0.7-1.1) |
| * The *p-*value was significant (*p* <0.05) | | |

**Table S6. Factors associated with multimorbidity in adjusted models**

| **Surveys** | **SADHS 2016** | **SABSSM 2017** | **NIDS 2017** |
| --- | --- | --- | --- |
| Increasing age | 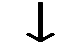 | 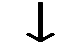 | 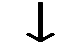 |
| Gender | - | 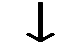 | - |
| Urban | - | 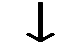 | - |
| Secondary education | - | - | 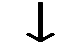 |
| Tertiary Education | 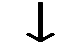 | 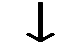 | - |
| Employment | 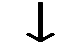 | 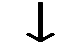 | - |
| Wealthiest quintile | - | - | 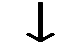 |
| Smoker | - | - | 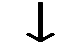 |
| Alcohol use | 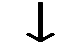 | - | - |
| High BMI | - | - | 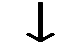 |
